# Reactivation of flagellar motility in demembranated *Leishmania* reveals role of cAMP in flagellar wave reversal to ciliary waveform

**DOI:** 10.1038/srep37308

**Published:** 2016-11-16

**Authors:** Aakash Gautam Mukhopadhyay, Chinmoy Sankar Dey

**Affiliations:** 1 Kusuma School of Biological Sciences, Indian Institute of Technology Delhi, Hauz Khas, New Delhi, India

## Abstract

The flagellum of parasitic trypanosomes is a multifunctional appendage essential for its viability and infectivity. However, the biological mechanisms that make the flagellum so dynamic remains unexplored. No method is available to access and induce axonemal motility at will to decipher motility regulation in trypanosomes. For the first time we report the development of a detergent-extracted/demembranated ATP-reactivated model for studying flagellar motility in *Leishmania*. Flagellar beat parameters of reactivated parasites were similar to live ones. Using this model we discovered that cAMP (both exogenous and endogenous) induced flagellar wave reversal to a ciliary waveform in reactivated parasites via cAMP-dependent protein kinase A. The effect was reversible and highly specific. Such an effect of cAMP on the flagellar waveform has never been observed before in any organism. Flagellar wave reversal allows parasites to change direction of swimming. Our findings suggest a possible cAMP-dependent mechanism by which *Leishmania* responds to its surrounding microenvironment, necessary for its survival. Our demembranated-reactivated model not only serves as an important tool for functional studies of flagellated eukaryotic parasites but has the potential to understand ciliary motility regulation with possible implication on human ciliopathies.

Leishmaniasis represents a group of geographically widespread diseases caused by different species of kinetoplastid parasites of genus *Leishmania*^1^. These parasites lead a digenetic life in two specific hosts, the sandfly, where they proliferate as motile, flagellated promastigotes and in mammals (including humans) where they invade utilizing their flagellum and then grow intracellularly^2^. The flagellum of trypanosomes like *Leishmania* is unusual in that it generates flagellar waves that propagate proximally from the tip (tip-to-base) that pulls the cell forward. These waves are interrupted by abrupt ‘cilia-like’ distally propagating waves (base-to-tip) enabling the cell to change direction in response to an obstacle^3^,^4^. The physics of parasite motility has received interdisciplinary focus due to its importance in host-parasite interactions^5^. However, till date flagellar motility and its regulation in *Leishmania* remains poorly understood despite the importance in its survival and infectivity.

In the sandfly host, the flagellum performs several attachment mechanisms that allow the passage of the promastigotes to anterior parts of the gut^6^,^7^. This ensures the proper positioning of the parasites to be transmitted by the insect’s bite and is possibly directed by chemotaxis^8^. Once the promastigotes are transferred to the mammalian host, the vigorous and unusual oscillations of flagellar tip invades the macrophages, reorienting the parasite and damaging the macrophage plasma membrane^2^. This uptake is highly reduced in immobile parasites^2^. The *Leishmania* flagellum thus, is a highly versatile organelle that exhibits intricate environment triggered responses far beyond simple fluid swimming behaviour^5^. Studies of the flagellar ultrastructure have been possible in the related trypanosome *Trypanosoma brucei* using RNAi techniques^9^ which is not possible in *Leishmania* spp^3^. except only in *L.* braziliensis^10^. In humans, defects in cilia cause a group of severe diseases called ciliopathies^11^. These defects constitute both structural defects as well as defects in the motility of the cilia. Eukaryotic parasites like trypanosomes have served as attractive models for the study of such genetic defects in humans with extensive research on structure and assembly of the cilia^12^. However, there is no suitable model till date for the study of the signalling and regulatory mechanisms of ciliary motility in ciliopathies.

Most of our current understanding of the regulatory mechanism controlling flagellar and ciliary beating come from detergent-extracted/demembranated, ATP reactivation studies in organisms like sperms of sea urchins, *Ciona*, dog, bull, flagella of *Chlamydomonas* and cilia of *Paramecium*^13^,^14^,^15^,^16^,^17^,^18^. In 1983, Alexander and Burns, reported their inability to reactivate demembranated *Leishmania* flagella^4^. Since then, no report on reactivation of *Leishmania* flagella has been published. The only trypanosome to receive attention using detergent-extracted models were the non-parasitic protozoa of genus *Crithidia*^19^,^20^. The fact that the flagellum is important for the viability and infectivity of pathogenic parasites like *Leishmania*, makes the information known on the non-parasitic species unsuitable to study its role in host-parasite relationships^3^. The absence of reports elucidating the control mechanism of flagellar motility in *Leishmania* led us to questions like: can the demembranated *Leishmania* flagella be reactivated? If so then would it be possible to tease out the regulatory mechanisms of flagellar motility? We presume that such a reactivated model will allow numerous studies in the future elucidating leishmanial flagellar motility and associated functionality for its survival and infection. Such a model would also have the potential to study the signalling pathways that possibly malfunction in ciliopathies.

## Results

### Reactivation of detergent-extracted/demembranated *L. donovani* parasites

**Figure 1:**
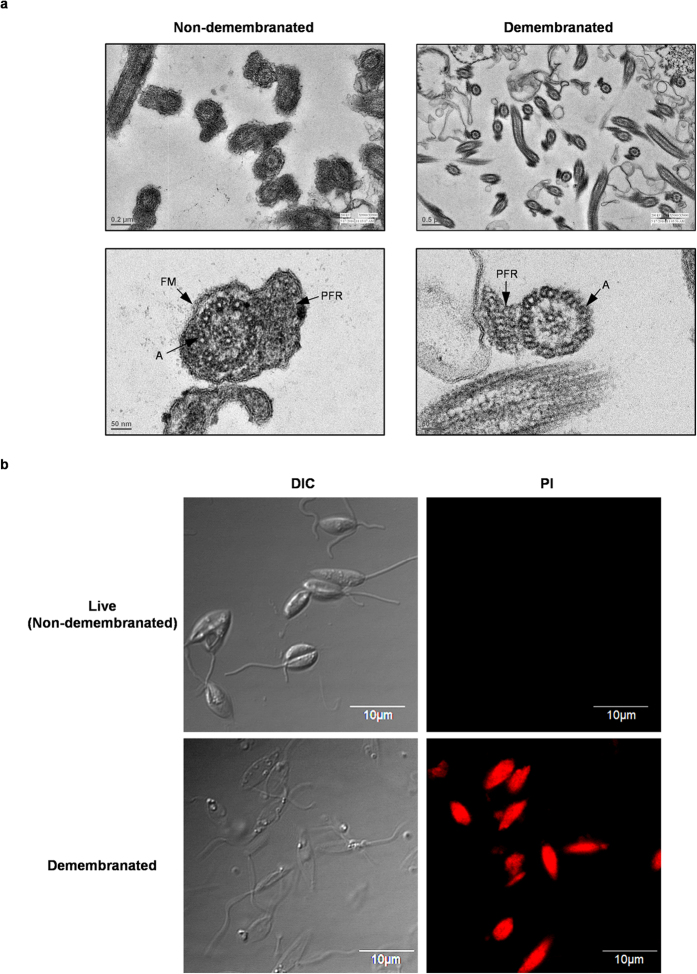
Permeabilization of *L. donovani* by 0.1% Triton demembranation. (**a**) Transmission electron micrographs (TEMs) of cross-sections of flagella of live and demembranated *L. donovani.* In live (non-demembranated) *Leishmania* intact outer flagellar membrane (FM) is visible. In demembranated *Leishmania* outer flagellar membrane (FM) is absent due to extraction with 0.1% Triton. (A) axoneme and (PFR) paraflagellar rod. (**b**) Confocal microscopy images of live (non-demembranated) and demembranated *L. donovani* treated with 15 μM propidium iodide (PI). Images were captured at 100X using an oil-immersion objective.

To reactivate flagellar motility of *L. donovani*, we first followed the protocol ideal for reactivation of bull sperm (basic protocol)^21^. Using the basic protocol we observed certain degree of reactivation of *L. donovani* however the beat parameters of the flagella were significantly reduced compared to live (non-demembranated) parasites (Table 1). Data clearly indicates that the reactivation condition for bull spermatozoa (basic protocol) was not suitable for optimal reactivation of *Leishmania* parasites. To improve the quality of reactivation and flagellar beat parameters specific to *Leishmania* we tested the effect of different concentrations of individual components of the reactivation medium on the beat frequency of the reactivated cells (Supplementary Fig. S1). Based on the conditions that led to improved beat frequency, we developed the following protocol namely *L eishmania* R eactivation P rotocol (LRP) (see Methods). Inductively Coupled Plasma Mass Spectrometry (ICP-MS) of the reactivation medium indicated the free calcium concentration to be between 2–4 × 10^−6^ M. For quantification of the flagellar beat parameters, videos of actively beating flagella of the reactivated cells were captured and analysed (see Methods). A small proportion (10–20%) of reactivated cells was observed to possess a ciliary waveform, generating base-to-tip waves. Table 1 shows a comparison between the flagellar beat parameters of live, LRP and basic protocol reactivated cells. LRP led to a significant increase in percent motility from 60.2% to 77.3% (*p* = 0.0212), beat frequency increased from 13.8 Hz to 19.2 Hz (*p* = 2.3E-09), amplitude improved from 1.88 μm to 2.02 μm (*p* = 0.035) and wavelength improved from 9.43 μm to 9.95 μm (*p* = 0.028). To test the efficiency of permeabilization by Trition demembranation, Transmission Electron Micrographs (TEMs) of cross-sections of flagella of live (non-demembranated) and detergent-extracted cells were performed. TEMs showed that the flagellar membrane of the detergent-extracted cells had been removed upon treatment with 0.1% Triton (Fig. 1a, upper panels). Figure 1a (lower panels) show that upon demembranation the flagellar membrane was efficiently removed without any structural damage to the axoneme. To further confirm the same we studied the uptake of the membrane impermeable fluorescent dye, propidium iodide (PI) in live and demembranated cells. Figure 1b shows that PI did not enter live cells. However, demembranated cells emitted a red fluorescence indicating uptake of PI. Thus, permeabilization of the membrane in the demembranation step was very efficient. In absence of ATP all demembranated cells remain immotile (Supplementary video 1, 2). However, motility was initiated almost immediately upon addition of ATP to demembranated cells (Supplementary video 3). To demonstrate that ATP was necessary to trigger motility in demembranated cells, video-micrographs were captured of immotile demembranated cells, to which ATP (final concentration 1.5 mM) was added. Motility was initiated once ATP diffused through the sample (Supplementary video 4). Both live (Supplementary video 5) and LRP reactivated cells (Supplementary video 6) showed a directional movement with planar beats of the flagella indicating tip-to-base wave propagation. The cells remained sufficiently motile for 10 minutes. However, possibly due to exhaustion of Mg-ATP and loss of structural integrity^21^ with time the percent motility dropped below 50% after 15 minutes. It is important to note that the proportion of motile cells varied from preparation to preparation. However, within one preparation, the results were consistent and reproducible.

**Figure 2:**
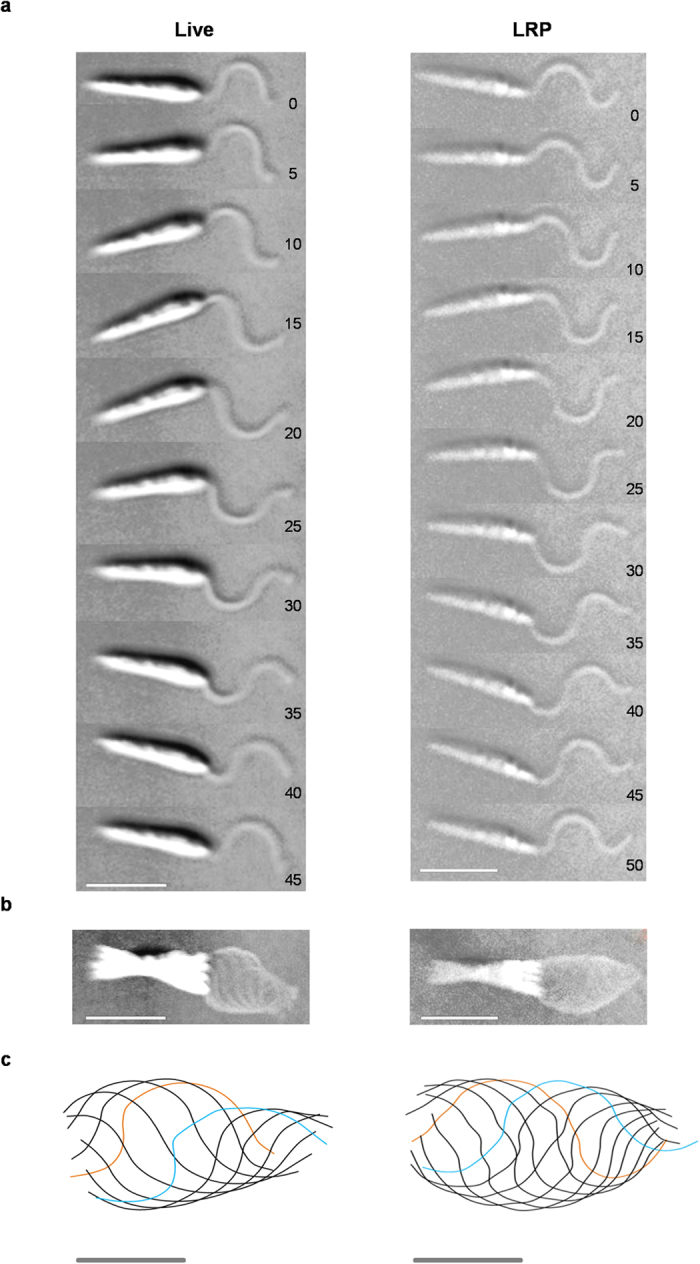
Illustrations of flagellar waveform of live and LRP reactivated *L. donovani.* (**a**) Image shows sequential stills from fast-capture videomicroscopy of live (beat frequency = 22.22 Hz) taken from video 5 and LRP reactivated (beat frequency 20 Hz) cells taken from video 6. Both cells generate waves proximally (tip-to-base). The cells complete one beat cycle in the times shown. Time intervals in milliseconds (ms) are shown on bottom right. Bar, 10 μm. (**b**) Image shows the superimposed flagellar waveforms of the corresponding images shown in ‘a’. Bar, 10 μm. (**c**) Superimposed traces of waveforms in ‘b’ using BohbohSoft. Red and blue lines correspond to first track and last track of the beat cycle respectively. Bar, 5 μm. Images of live and LRP reactivated cells were taken as representative of cells that were quantified and summarized in Table 1.

The flagellar wave of live *L. donovani* was symmetrical, planar and originated from the tip of the flagella (Fig. 2a–c: left side panels and Supplementary video 5). Similar to the live cells, reactivated cells in LRP demonstrated a symmetrical, planar flagellar wave (Fig. 2a–c: right side panels and Supplementary video 6). Supplementary Fig S2 shows that the beat parameters of reactivated parasites in LRP possessed similar relationships between the beat parameters of live parasites.

One of the notable advantages of our technique is that it allows both demembranation and reactivation in the same medium. This ensures that the cellular contents/cytosol of the extracted cells remains in the same solution and hence mediates their effects. To determine whether presence of detergent and cytosol were necessary for flagellar motility of reactivated parasites, we reactivated demembranated cells in absence of detergent and cytosol (see Methods). Supplementary Table S1 shows a comparative between the beat parameters of live, and cells reactivated in LRP with and without detergent and cytosol. Our results indicate that there was no significant effect on beat parameters upon removal of detergent and cytosol from the reactivation medium. Therefore, we performed our reactivations in presence of both detergent and cytosol. This was also expected to maintain the cellular health/integrity due to lesser handling of the cells before subjecting them to reactivation. The classical experiments by Lindemann in 1978 were also performed in presence of detergent and cytosol without affecting the overall motility^17^. Our results confirm successful development of a method of demembranation and reactivation of *Leishmania* that possessed similar flagellar motility parameters as that of the live ones.

### Effect of exogenous cAMP on motility and flagellar waveform of reactivated parasites

**Figure 3:**
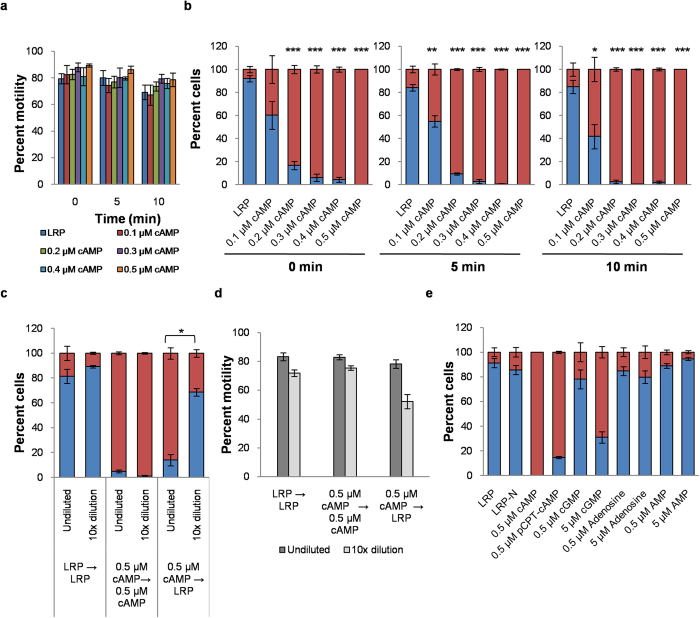
Effect of exogenous cAMP on LRP reactivated *L. donovani.* (**a**) Effect of increasing concentrations of exogenous cAMP on percent motility of reactivated cells with time. (**b**) Effect of increasing concentrations of exogenous cAMP on the waveform proportions of LRP reactivated *L. donovani*. Cells were reactivated in LRP in presence of increasing concentrations of cAMP and the proportions of motile cells with flagellar and ciliary waveforms were quantified over 0, 5 and 10 min. (**c**) Reversibility of the effect of cAMP on waveform of LRP reactivated parasites. LRP → LRP denotes LRP reactivated parasites were diluted 10 times in LRP. 0.5 μM cAMP → 0.5 μM cAMP denotes LRP reactivated parasites in presence of 0.5 μM cAMP were diluted 10 times in LRP containing 0.5 μM cAMP. 0.5 μM cAMP → LRP denotes LRP reactivated parasites in presence of 0.5 μM cAMP were diluted 10 times in LRP (without cAMP). (**d**) Percent motility of cells reactivated in LRP (with or without 0.5 μM cAMP) and then diluted 10 times in ‘c’. (**e**) Chemical specificity of LRP reactivated cells towards cAMP. Distribution of reactivated cells with flagellar and ciliary waveforms plotted at 0 min time point in presence of cAMP, cGMP, pCPT-cAMP, AMP and adenosine. LRP-N denotes reactivation in presence of 10 mM NH_4_OH. Adenosine was dissolved in NH_4_OH. Red denotes proportion of motile cells with ciliary waveform. Blue denotes proportion of motile cells with flagellar waveform. All experiments were performed three times. Values are mean ± SEM. *p < 0.05; **p < 0.01; ***p < 0.001.

We investigated the effects of cAMP on *Leishmania* flagellar motility using the reactivated model. Increasing concentrations of cAMP (0.1 μM to 0.5 μM) was added exogenously over a time period of 0 (images were captured immediately after ATP addition and had a delay time of 30–40 sec), 5 and 10 min to determine whether cAMP had any effect on the percent motility over time. Exogenous addition of increasing concentrations of cAMP to LRP had no effect in the percent motility (Fig. 3a). Beyond 10 min percent motility dropped rapidly when the cells became jittery with attenuated beat parameters. Data shows no significant change in percent motility upon addition of cAMP over a time period of 10 min. Our results show that exogenous cAMP had no effect on overall percent motility unlike reports in spermatozoa^17^,^18^,^22^,^23^,^24^,^25^.

Next we investigated whether cAMP had any effect on the waveform of the flagella of reactivated *L. donovani*. To address this issue, the same video-micrographs taken for the time-course experiment (Fig. 3a) were used to quantify the proportions of cells possessing either flagellar or ciliary waveforms by assaying for direction of wave propagation and wave asymmetry. At 0 min, the proportion of motile cells with flagellar waveform was 92.03 ± 2.71% (Fig. 3b). To our surprise, with increasing concentrations of exogenous cAMP we observed the proportions of motile cells beating with ciliary (reverse) waveform increased with time (Fig. 3b). The effect of exogenous cAMP on wave reversal was dose-responsive. Addition of 0.5 μM cAMP immediately led to almost 100% cells to beat with ciliary waves (*p* = 4.5E-06). Our results suggest that 0.5 μM cAMP was sufficient to induce almost complete wave reversal almost immediately after addition to reactivated flagella of *L. donovani.* Moreover, for lower concentrations of cAMP, the effect was gradual (Fig. 3b).

To test whether the effect of cAMP on flagellar wave reversal of reactivated *Leishmania* was reversible, we reactivated the parasites in presence of 0.5 μM cAMP (which induces almost complete reversal) and after ~1 min diluted the cAMP to 0.05 μM by adding LRP medium. Video-micrographs of reactivated parasites were captured before and after dilution. In presence of 0.5 μM cAMP, ~90% cells were beating with ciliary waveform. However, when cAMP concentration was diluted 10 times, cells beating with ciliary waveform were only ~30% (*p* = 0.018) (Fig. 3c). Thus the effect of cAMP on the flagellar waveform of reactivated parasites was reversible. Dilution by 10 times had no significant effect on the percent motility of cells (Fig. 3d).

To test the specificity of the response of reactivated *L. donovani* to cAMP we investigated the effect of addition of molecules similar to cAMP on reactivated cells (Fig. 3e). cGMP could not induce equal wave reversal effects as cAMP (Fig. 3e). It has been previously reported that the anti-proliferative effects mediated by cAMP and its analogs in *T. brucei* are not due to cAMP itself but by hydrolysis products of cAMP, like AMP and adenosine^26^. To address this issue we used a known phosphodiesterase (PDE) resistant analog, pCPT-cAMP^27^,^28^. 0.5 μM pCPT-cAMP like 0.5 μM cAMP induced almost 100% cilia-like beating in reactivated parasites. Addition of the cAMP hydrolysis products AMP and adenosine did not significantly alter the proportions of cells beating with ciliary or flagellar waveforms compared to LRP reactivated cells. Thus, we concluded that the effects of cAMP on reactivated cells in LRP was highly specific and due to cAMP itself and not its breakdown products.

**Figure 4:**
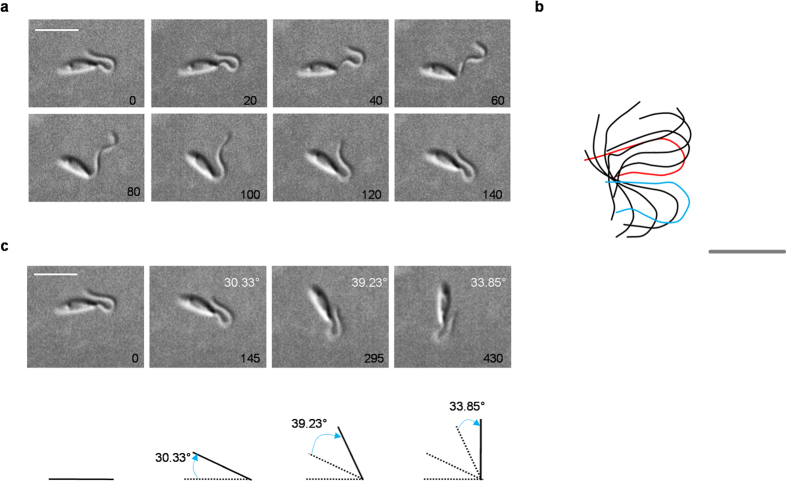
Ciliary (base-to-tip) waveform in LRP reactivated *L. donovani* in presence of 0.5 μM cAMP. (**a**) A montage of stills showing one complete ciliary beat of a LRP reactivated cell (Beat frequency = 6.89 Hz) with 0.5 μM cAMP taken from video 7. Camera exposure was 5 milliseconds (ms) however, we displayed the images taken after every 20 ms. Time intervals (ms) are shown on bottom right. Bar, 10 μM. Images were taken as representatives of cells that were quantified and summarized in Table 2. (**b**) Ciliary waveforms of ‘a’ were tracked and superimposed using BohBohSoft. Red and blue lines correspond to first track and last track of the beat cycle respectively. Bar, 5 μm. (**c**) Upper panel shows stills of the cell orientation at the beginning of 4 consecutive beat cycles. The degree of change in direction per beat are shown on top right (white). Bar, 10 μm. Bottom panel displays a line drawing representation of corresponding stills above. Solid line denotes present position of the cell. Dotted line denotes previous position(s). Blue arrow represents the angle by which the cell reorients itself in one beat cycle.

The ciliary waveform of reactivated *L. donovani* in presence of 0.5 μM cAMP possessed a distinct power stroke followed by an atypical recovery stroke. The cells lacked direction, with no translational motion and kept turning around a point with planar waves (Fig. 4a and Supplementary video 7). The waveform was highly asymmetric, with a beat frequency of about one fifth of that of the flagellar wave (Fig. 4b and Table 2). With each beat the cells reoriented by ~30° (Fig. 4c). This leads to a whiplash-like waveform while the cell keeps turning or tumbling. The cilia-like wave initiated from the base of the flagella and traversed towards the tip and is identical to that observed in live cells (Supplementary video 8). Once the live cell has altered its direction, flagellar (tip-to-base) beating takes over. Table 2 summarizes the beat parameters of reactivated cells with ciliary waveform of beating flagella induced by 0.5 μM cAMP. The beat parameters of the ciliary waveform were highly attenuated in comparison to flagellar beating. We concluded that exogenous cAMP induced a ciliary waveform to previously flagellar waveform in reactivated *L. donovani* and therefore was responsible for change in direction of wave propagation (converts previously tip-to-base waves to base-to-tip waves). This response to cAMP was dose-responsive, reversible and highly specific.

### Effect of endogenous cAMP on motility and flagellar waveform of reactivated parasites

**Figure 5:**
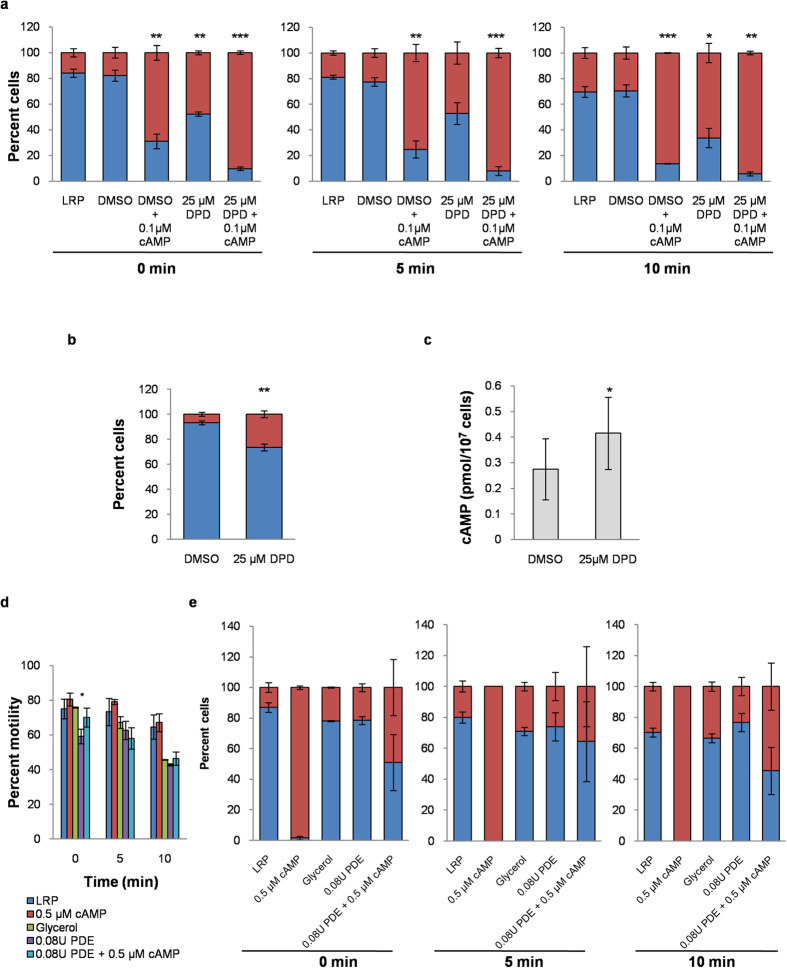
Effect of endogenous cAMP levels on LRP reactivated *L. donovani*. (**a**) Effect of dipyridamole (DPD) on proportions of reactivated cells with flagellar and ciliary beats. 0.1% DMSO control was maintained as DPD was dissolved in DMSO. Proportions of cells with ciliary and flagellar waveforms were quantified for each sample. (**b**) Effect of pre-treatment of live parasites with DPD. Parasites were pretreated with 25 μM DPD for 1 hour and subsequently reactivated in LRP without any exogenous cAMP. Proportions of cells with ciliary and flagellar waveforms were quantified for each sample. 1% DMSO control was maintained. (**c**) Intracellular cAMP concentration of live *L. donovani* parasites treated with or without 25 μM DPD for 1 hour. (**d**) Effect of endogenous cAMP levels on motility using phosphodiesterase enzyme (PDE) in LRP with time. A glycerol control was maintained as the enzyme was dissolved in 50% glycerol. **(e)** Effect of PDE on proportions of reactivated cells with flagellar and ciliary beats. Red denotes proportion of motile cells with ciliary waveform. Blue denotes proportion of motile cells with flagellar waveform. All experiments were performed three times. Values are mean ± SEM. *p < 0.05; **p < 0.01; ***p < 0.001.

In *Leishmania* only cAMP-dependent phosphodiesterase enzyme (PDE) activity has been identified^29^,^30^. Dipyridamole (DPD) is one of the few effective inhibitors of leishmanial PDEs with an IC50 of 29 μM^27^,^31^. We tested the effect of 6.25, 12.5, 25, 50 and 100 μM DPD on the proportions of flagellar and ciliary cells. DPD did not have any significant effect on percent motility (Supplementary Fig. S3a). 25 μM was the lowest concentration at which maximum wave reversal of 50–60% was observed (Supplementary Fig. S3b). Therefore 25 μM DPD was effective in inhibiting the endogenous PDEs of *Leishmania*. Using this concentration we investigated whether DPD and exogenous 0.1 μM cAMP had a synergistic or additive effect on reversal of wave direction. At 0 min, 0.1 μM cAMP and 25 μM DPD alone caused approximately 70% and 47% of motile cells to beat with ciliary waveform respectively (Fig. 5a). When added together, ~90% of motile cells were ciliary with increase in ciliary waveform by 30.59 ± 8.17% (*p* = 0.022) and 89.01 ± 2.86% (*p* = 3.3E-05) over 0.1 μM cAMP and 25 μM DPD respectively. No significant change in percent motility was observed (Supplementary Fig. S3c). The presence of soluble adenylate cyclase has been previously reported in *Leishmania*^32^ which possibly remains active in solution even after demembranation. Data suggests that pharmacological inhibition of endogenous cAMP-dependent PDEs by DPD led to an increase in cAMP levels with time which led to an increase in the number of cells beating with ciliary waveform. Endogenous levels of cAMP are maintained by enzyme activity of adenylate cyclases (both transmembrane and soluble enzymes) and PDEs. Possibly, inhibition of endogenous PDEs shifted the endogenous cAMP balance in favour of soluble adenylate cyclases that produce cAMP.

To further confirm our findings we pre-treated live *Leishmania* parasites with 25 μM DPD for 1 hour, after which the cells were washed once in HBSSDG (see Methods) and subsequently reactivated in LRP without addition of exogenous cAMP (final cell concentration ~8 × 10^3^ cells/μl). Proportions of cells beating with ciliary waveform increased significantly with 25 μM DPD pre-treatment (*p* = 0.0033) (Fig. 5b). No significant change in percent motility was observed (Supplementary Fig. S3d). When endogenous cAMP levels of pre-treated live cells were measured, a significant ~50% increase in cAMP concentration (*p* = 0.025) was observed in live cells pre-treated with 25 μM DPD compared to control cells (Fig. 5c). Our results suggest that inhibition of endogenous PDEs by DPD led to elevated endogenous cAMP levels in the treated live cells which when reactivated in absence of exogenous cAMP induced ciliary beating.

To investigate the role of endogenous cAMP in regulating flagellar motility, we studied the effect of 0.08 U/ml PDE on motility and waveform of reactivated *L. donovani*. A significant decrease in percent motility was observed upon PDE addition at the 0 min time point (*p* = 0.017) (Fig. 5d). However, beyond the immediate drop in percent motility, there was no significant change in 5 and 10 min time points compared to control (Fig. 5d). Addition of PDE to reactivated flagella had no significant effect on the proportions of flagellar and ciliary beating cells (Fig. 5e). To confirm that the enzyme was indeed active, cAMP was added exogenously. The concentration of 0.5 μM cAMP added exogenously was sufficient to induce complete wave reversal at 0 min time point. However, after 10 minutes 54.52 ± 15.73% of the cells were beating with the ciliary waveform, indicating that the enzyme was active. The initial drop in percent motility suggests the possibility that the basal endogenous cAMP levels are necessary to maintain overall reactivated motility. However it is interesting that PDE alone did not alter the proportions of motile parasites beating with ciliary and flagellar waveforms. Data further confirms that wave reversal by cAMP occurs only when cellular cAMP concentration rise above the endogenous level. Decrease in endogenous cAMP concentration in reactivated parasites had no effect on proportions of flagellar and ciliary cells.

### Effect of Protein Kinase A inhibitors on flagellar waveform

**Figure 6:**
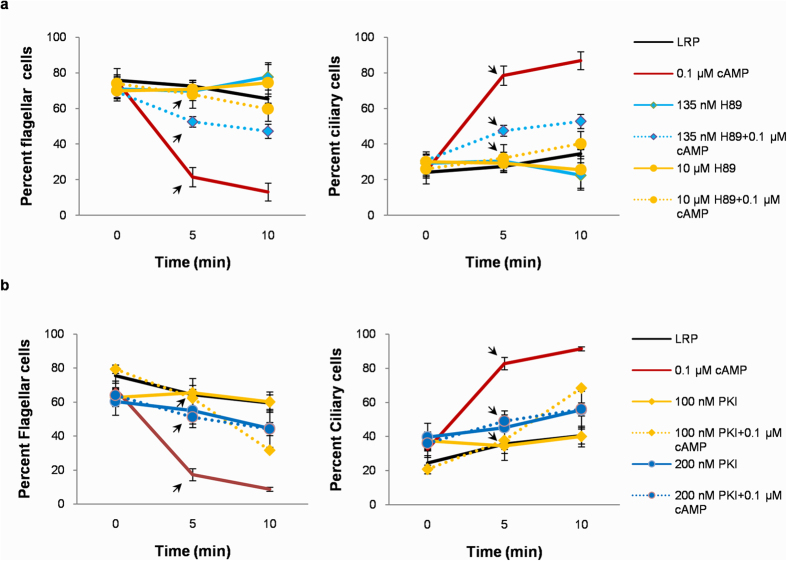
Effect of PKA inhibition on the distribution of reactivated *L. donovani* with flagellar and ciliary waveforms. (**a**) Effect of H89 on proportions of reactivated cells with flagellar and ciliary beats. Cells were reactivated in LRP in presence of increasing concentrations of H89 and the proportions of motile cells with flagellar and ciliary waveforms were quantified over 0, 5 and 10 mins. (**b**) Effect of PKI on proportions of reactivated cells with flagellar and ciliary beats. Cells were reactivated in LRP in presence of increasing concentrations of PKI and the proportions of motile cells with flagellar and ciliary waveforms were quantified over 0, 5 and 10 mins. In ‘a’ and ‘b’, 0.1 μM cAMP was added to reactivated cells at 5 min time point shown with black arrows. All experiments were performed three times. Values are mean ± SEM.

In *Leishmania*, cAMP-dependent protein kinase A (PKA) regulatory subunit has been identified, over expression of which resulted in increase in percent motile cells compared to control parasites^33^. We used two PKA inhibitors H89 and Protein kinase A inhibitor fragment 6–22 amide (PKI) to further investigate a possible mechanism by which cAMP mediates its effect on the wave direction in demembranated *Leishmania.* Both inhibitors are known to inhibit leishmanial PKA^34^. Two concentrations of H89, 135 nM which is its IC_50_ and 10 μM, a concentration which has been reported to be effective in inhibiting leishmanial PKA were used^35^,^36^. Figure 6a shows that neither 135 nM nor 10 μM H89 had any effect on the proportions of flagellar and ciliary cells as compared to LRP. Addition of 0.1 μM cAMP to LRP at 5 min immediately dropped the proportions of flagellar cells by 70.29 ± 7.47% (*p* = 0.0014) and at 10 min by 79.98 ± 7.68% (*p* = 0.0008). When 0.1 μM cAMP was added to cells pre-incubated for 5 min with 135 nM H89 in LRP, proportions of flagellar cells decreased by only 24.54 ± 4.35% (*p* = 0.045) at 5 min and by 39.09 ± 5.13% (*p* = 0.02) at 10 min compared to 135 nM H89 treated cells in LRP alone. Interestingly, when 0.1 μM cAMP was added to cells pre-incubated with 10 μM H89 for 5 min in LRP, there was no significant change in the proportions of cells beating with flagellar and ciliary waveforms even at 10 min time point compared to LRP. PKI (6–22) amide has been shown to be an effective PKA inhibitor at a concentration of 100 nM^37^. It is highly specific to PKA without inhibiting other kinase activity like protein kinase G at concentrations higher than 100 nM^38^. Figure 6b shows addition of 100 nM PKI to LRP did not alter the proportions of flagellar and ciliary cells as compared to LRP. Addition of 0.1 μM cAMP to LRP at 5 min immediately dropped the proportions of flagellar cells by 73.19 ± 5.57% (*p* = 1.8E-06) and at 10 min by 85.52 ± 2.04% (*p* = 1.1E-06). When 0.1 μM cAMP was added to cells pre-incubated for 5 min with 100 nM PKI in LRP there was no significant change in proportions of flagellar cells at 5 min time point compared to cells just preincubated with 100 nM PKI. However, proportions of flagellar cells decreased by 47.33 ± 1.67% (*p* = 0.009) at 10 min time point compared to 100 nM PKI treated cells in LRP alone. Doubling the PKI concentration to 200 nM PKI alone did not alter the proportions of flagellar and ciliary cells. No significant change in flagellar and ciliary cells was observed upon addition of 0.1 μM cAMP to cells in 200 nM PKI at 5 and 10 min time points as compared to 200 nM PKI alone. PKA inhibition by H89 and PKI resisted the wave reversal effects of exogenous cAMP on reactivated parasites. Therefore, we concluded that cAMP mediates its effects of flagellar wave reversal via its downstream kinase PKA.

## Discussion

**Figure 7:**
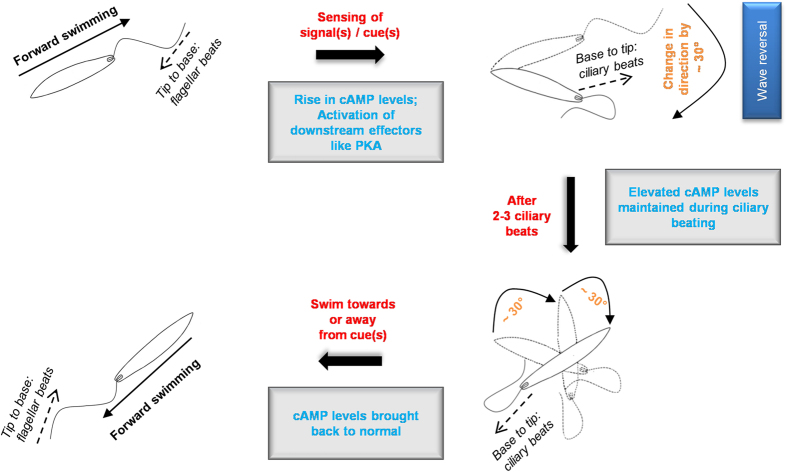
Diagrammatic representation of possible regulation of motility by cAMP mediated wave reversal in *L. donovani* promastigotes. A cAMP-dependent signal transduction cascade is triggered when the flagella of a forward swimming parasite encounters any signal(s)/cue(s) (e.g. physical obstruction or osmotic/chemical gradient). The elevated cAMP levels allow the base-to-tip ciliary beating to take over tip-to-base flagellar beating. Ciliary beating allows the cell to alter its direction, after which tip-to-base beating is restarted. Solid arrows denote direction of swimming. Dotted arrows denote direction of wave propagation of flagella. Dotted outline of *Leishmania* denotes previous position. Filled outline of *Leishmania* denotes current position.

Our study has two major contributions to the understanding of flagellar motility of the protozoan parasite *Leishmania donovani.* Firstly, we have successfully reactivated demembranated flagella of *Leishmania.* After the study by Alexander and Burns in 1983 where they report their inability to reactivate *Leishmania* flagella, to our knowledge there has been no publication till date reporting its successful reactivation. We believe that our success in reactivating the *Leishmania* flagella presents it as an attractive model suitable for investigating flagellar motility regulation in parasitic protozoa. Secondly, we discovered a novel role of cAMP in regulating wave reversal in reactivated flagella. Such an effect of cAMP on flagellar waveform and direction of wave propagation has never been reported before in any organism. Wave reversal in *Leishmania* appears analogous to bacterial tumbling behaviour during chemotaxis^39^. Previously base-to-tip ciliary beating has been reported in *C. oncopelti*^19^,^20^ and *C. fasciculata, C. deanei* and *L. major*^3^. It has been speculated that this beating might constitute an obstacle-avoidance response. The molecular mechanism of how the flagellum of trypanosomes sense and respond to changes in surrounding microenvironment remains unresolved^40^. Figure 7 shows a diagrammatic representation of the possible mechanistic events that take place when a *Leishmania* parasite encounters any change in its immediate surrounding environment^8^,^41^.

Recently CRISPR-Cas9 system has shown promise for functional studies in *Leishmania*^42^. However, functional pleiotropy of the flagellum makes it difficult to interpret such mutant phenotypes complicating the distinction between direct, indirect, and stage-specific effects on cellular function^5^,^43^,^44^. To address such issues, detergent-extracted models are an option for studying differences in the function of the flagella under standardized assay conditions^21^. CRISPR-Cas9 coupled with our reactivated model in *Leishmania* bears immense potential to perform functional studies on the axoneme. Flagellar gene candidates in *T. brucei* have been identified to have human homologs that map to loci responsible for ciliopathies^45^. Therefore our findings may have impact on future research on human ciliary diseases.

Flagellar wave reversal has been described previously in the detergent-extracted non-parasitic trypanosome *Crithidia* by calcium^19^,^20^. Flagellar waves were observed at calcium concentrations < 10^−7^ M. However, we observed flagellar waves identical to live parasites in presence of > 10^−6^ M free calcium. This substantiates high species diversity within parasitic and non-parasitic trypanosomes. This signifies the necessity of understanding the molecular and biochemical mechanism that regulates flagellar beating in human parasitic trypanosomes like *Leishmania*. Studies of the flagellar beat in *Trypanosoma* is hampered by the attachment of the flagella to its cell body^3^. Our detergent-extracted ATP reactivated model of *Leishmania* overcomes such caveats for studies on flagellar beat and its controlling mechanisms in parasitic protozoa.

The cyclic nucleotide signalling pathways in trypanosomatids are significantly different from their mammalian hosts^46^. In *T. brucei*, cAMP regulates social motility^47^. A cAMP signalling pathway in *T. brucei* flagellar membrane has been implicated in its virulence^48^. The fact that cAMP levels regulate social motility in *T. brucei*, coupled with our findings of cAMP and its effect on flagellar wave reversal in *L. donovani* makes us ask questions like, are these unique effects of cAMP necessary for the parasitic lifestyle of these trypanosomes? Apart from tactic roles, does flagellar wave reversal have any implication of mammalian tissue invasion? Further studies on signalling pathways and their effects on motility can only answer these questions.

## Methods

### Parasite Cell Culture

Wild-type *Leishmania donovani* promastigotes (AG83) were a kind gift from Dr. Amitabha Mukhopadhyay (National Institute of Immunology, India). Promastigotes were grown in fresh Medium 199 containing 10% (v/v) heat inactivated fetal bovine serum (Life Technologies, USA) and 50 μg/ml gentamicin (Himedia, India) for 4 days at 24 °C before further experimentation. Cells were seeded at 0.5 × 10^6^ cells/ml and subcultured every four days. Experiments were performed on cells in mid-logarithmic growth phase, as reported previously from our laboratory^49^.

### Reactivation of Detergent-extracted/Demembranated *Leishmania donovani*

20 × 10^6^ *L. donovani* promastigotes were washed twice with Hank’s Balanced Salt Solution + 1% D-glucose (HBSSDG) by gentle centrifugation at 600 × g at 20 °C for 5 min^4^ and the washed pellet was resuspended in 200 μl HBSSDG. For each reactivation, 20 μl of washed cells was added to 250 μl reactivation mixture (final cell concentration ~4 × 10^3^ cells/μl) in a glass bottom petridish composed of 24 mM potassium glutamate, 140 mM sucrose, 20 mM Tris-Cl, 1 mM DTT, 0.1% Triton X-100, 1.5 mM MgSO_4_, 2 mM EGTA and 0.25 mM EDTA (pH 7.8), demembranated for ~30 seconds, followed by adding 1.5 mM ATP to reactivate the flagella. This protocol was named as *L eishmania* R eactivation P rotocol (LRP). The samples were immediately observed under a microscope and video-micrographs were captured as mentioned below. All experiments were performed at room temperature (~24 °C).

For reactivation in absence of detergent and cytosol: 20 × 10^6^ *L. donovani* promastigotes were washed twice with HBSSDG by gentle centrifugation at 600 × g at 20 °C for 5 min and the washed pellet was resuspended in 100 μl HBSSDG. For demembranation, 20 μl of washed cells was added to 130 μl demembranation solution, 24 mM potassium glutamate, 140 mM sucrose, 20 mM Tris-Cl, 1 mM DTT, 0.1% Triton X-100, 1.5 mM MgSO_4_, 2 mM EGTA and 0.25 mM EDTA (pH 7.8) and demembranated for ~30 seconds. For reactivation, 50 μl of demembranated cells were added to 250 μl reactivation solution, 24 mM potassium glutamate, 140 mM sucrose, 20 mM Tris-Cl, 1 mM DTT, 1.5 mM MgSO_4_, 2 mM EGTA and 0.25 mM EDTA, 1.5 mM ATP (pH 7.8). Final cell concentration ~2 × 10^3^ cells/μl. Video-micrographs of reactivated cells were captured as mentioned below.

### Fast-Capture Videomicroscopy

For video capturing of live cells, 20 × 10^6^ *L. donovani* promastigotes were washed twice with HBSSDG by gentle centrifugation at 600 × g at 20 °C for 5 min and the washed pellet was resuspended in 200 μl HBSSDG. 20 μl of washed cells were added to 250 μl HBSSDG (final cell density ~4 × 10^3^ cells/μl) in a glass bottom petridish (Greiner BioOne) and videos were recorded. The motility parameters determined on these samples served as live control against reactivated motility.

Video-micrographs of live and reactivated *Leishmania* were captured on a Nikon Eclipse Ti-E microscope attached to an Andor SCMOS Camera Zyla (USA) using Nikon NIS Basic Research software 4.13, 64 bit. All videos were captured at 200 frames per second for a duration of 2 s (~400 frames) with camera resolution 512 × 512 pixels, 16 bit-depth with an exposure time of 5 ms for waveform analysis. Differential interference contrast illumination was used at a magnification of 40X (numerical aperture 0.60). For percent motility calculation, videos were captured between 15–20 frames per second for duration of 2 s with camera resolution 2560 × 2160 pixels, 16 bit-depth with an exposure time of ~10 ms at a magnification of 40X. A minimum of 50 cells were counted per sample per experiment. All cells (live or reactivated) with actively beating flagella were considered motile. Percent motility denotes the number of motile cells out of total number cells quantified.

### Image Processing

All pre- and post-analysis image processing were performed using Nikon NIS Basic Research software 4.13, 64 bit. Flagellar tracking was performed using image analysis software BohBohSoft (BohBohSoft, Japan).

### Measurement and Calculation of Beat Parameters

Measurements of beat parameters were performed using NIS 4.13 software. The mean amplitude and wavelength for the beats of individual cells were determined by using the “2 points” tool under “Annotations and Measurements” tab for measuring the length between the minima and maxima of processed images of flagella. The beat frequency was determined by measuring the period of 3 beats per individual cell and taking the average of the 3 beats. The wave propagation speed was calculated as the product of the wavelength and beat frequency. The change in direction of ciliary cells was determined using the “Measure free angle” tool to measure the angle between its position at the beginning and end of a complete beat. The flagellum length was measured using the “Polyline” tool.

### Transmission Electron Microscopy (TEM)

25 ml parasite culture was grown to a density of ~20 × 10^6^ cells/ml to obtain a reasonably large pellet for TEM. The culture medium was washed twice with HBSSDG at 800 × g for 5 min. For live (non-demembranated) cells, the final washed pellet was fixed in 2.5% glutaraldehyde and 2% paraformaldehyde in phosphate buffered saline for 2 hours at room temperature followed by 4 °C overnight. For demembranated cells, the washed pellet was demembranated in LRP medium containing 0.1% Triton X-100 (without ATP) for ~30 seconds, after which the demembranated cells were pelleted down and fixed as mentioned above. Before fixation, both live and demembranated cells were checked under the microscope to ensure proper removal of media that interfere with TEM imaging, and to ensure adequate demembranation of the treated cells. Blocks were prepared, sectioned and stained at SAIF, AIIMS, New Delhi. Electron micrographs were captured on a Tecnai G2 20 S-Twin TEM (FEI) and captured with Gatan, 4 k × 4 k camera at SAIF, AIIMS.

### Confocal Laser Scanning Microscopy

30 × 10^6^ parasite cells were washed twice in HBSSDG as mentioned previously. The washed pellet was either resuspended in 500 μl HBSSDG containing 15 μM propidium iodide (PI) or in 500 μl demembranation medium containing 15 μM PI and incubated for 5 min at room temperature. After incubation, the dye was removed by washing twice in PBS and the washed cells (live and demembranated) were visualized by confocal microscopy (Olympus Fluoview 1000, Japan) at a magnification of 100X oil-immersion objective.

### Inductively Coupled Plasma Mass Spectrometry (ICP-MS)

For measurement of free calcium ion concentration in LRP medium, the reaction mixture was up-scaled 20 times. The solution was filtered through a 0.2 μm syringe filter followed by acidification by ultrapure nitric acid. The free calcium ion present was measured using the Agilent ICP-MS 7900 with Ultra High Matrix Induction (UHMI) in CRF, IIT Delhi.

### Assay of Intracellular cAMP

10 × 10^6^ parasites were washed as mentioned above, and the pellet was resuspended in 25 μM DPD in HBSSDG (treated) or HBSSDG + DMSO (control) and incubated for 1 hour. The cells were then washed once, the washed pellet was demembranated for ~30 s in LRP medium, and then centrifuged at 8000 × g, 5 min. cAMP concentrations in the supernatant was determined using the cAMP EIA assay kit (Sigma Aldrich, USA) with an acetylation step for higher sensitivity according to the instructions of the manufacturer.

### Statistical Analysis

All independent experiments were performed at least three times. The data are expressed as mean ± SEM. For comparison of two groups, *p*-values were calculated by two-tailed Student’s *t*-test. In all cases *p* < 0.05 was considered to be statistically significant.

### Reproducibility of experiments

Figure-wise samples size (number of cells quantified) is mentioned as follows:

Figure 3a: LRP 0 min (318), 5 min (336), 10 min (285); 0.1 μM cAMP 0 min (356); 5 min (220); 10 min (236); 0.2 μM cAMP 0 min (337), 5 min (329), 10 min (379); 0.3 μM cAMP 0 min (423), 5 min (328), 10 min (318); 0.4 μM cAMP 0 min (345), 5 min (384), 10 min (298); 0.5 μM cAMP 0 min (364), 5 min (386), 10 min (338). Figure 3b: LRP 0 min (248), 5 min (263), 10 min (192); 0.1 μM cAMP 0 min (292); 5 min (161); 10 min (157); 0.2 μM cAMP 0 min (280), 5 min (252), 10 min (277); 0.3 μM cAMP 0 min (369), 5 min (265), 10 min (251); 0.4 μM cAMP 0 min (278), 5 min (304), 10 min (229); 0.5 μM cAMP 0 min (325), 5 min (331), 10 min (266). Figure 3c: LRP → LRP undiluted (238), 10x dilution (269); 0.5 μM cAMP → 0.5 μM cAMP undiluted (266), 10x dilution (174); 0.5 μM cAMP → LRP undiluted (227), 10x dilution (154). Figure 3d: LRP → LRP undiluted (286), 10x dilution (379); 0.5 μM cAMP → 0.5 μM cAMP undiluted (321), 10x dilution (231); 0.5 μM cAMP → LRP undiluted (289), 10x dilution (290). Figure 3e: LRP (231), LRP-N (102), 0.5 μM cAMP (297), 0.5 μM pCPT-cAMP (246), 0.5 μM cGMP (223), 5 μM cGMP (245), 0.5 μM Adenosine (115), 5 μM Adenosine (105), 0.5 μM AMP (209), 5 μM AMP (142).

Figure 5a: LRP 0 min (141), 5 min (143), 10 min (152); DMSO 0 min (179), 5 min (142), 10 min (111); DMSO + 0.1 μM cAMP 0 min (269), 5 min (162), 10 min (140); 25 μM DPD 0 min (162), 5 min (172), 10 min (136); 25 μM DPD + 0.1 μM cAMP 0 min (222), 5 min (187), 10 min (148). Figure 5b: DMSO (152), 25 μM DPD (125). Figure 5d: LRP 0 min (293), 5 min (291), 10 (301); 0.5 μM cAMP 0 min (268), 5 min (289), 10 min (320), Glycerol 0 min (281), 5 min (241), 10 min (194); 0.08U PDE 0 min (359) 5 min (304), 10 min (286), 0.08U PDE + 0.5 μM cAMP 0 min (216) 5 min (135), 10 min (117). Figure 5e: LRP 0 min (223), 5 min (214), 10 min (198); 0.5 μM cAMP 0 min (211), 5 min (227), 10 min (214); Glycerol 0 min (213), 5 min (163.5), 10 min (89); 0.08U PDE 0 min (219), 5 min (192), 10 min (122); 0.08U PDE + 0.5 μM cAMP 0 min (216), 5 min (135), 10 min (117).

Figure 6a: LRP 0 min (158), 5 min (116), 10 min (123); 0.1 μM cAMP 0 min (183), 5 min (198), 10 min (161); 135 nM H89 0 min (125), 5 min (91), 10 min (79); 135 nM H89 + 0.1 μM cAMP 0 min (154), 5 min (125), 10 min (149); 10 μM H89 0 min (134), 5 min (106), 10 min (81); 10 μM H89 + 0.1 μM cAMP 0 min (127), 5 min (107), 10 min (124). Figure 6b: LRP 0 min (292), 5 min (223), 10 min (207); 0.1 μM cAMP 0 min (277), 5 min (388), 10 min (226); 100 nM PKI 0 min (112), 5 min (83), 10 min (64); 100 nM PKI + 0.1 μM cAMP 0 min (89), 5 min (112), 10 min (104); 200 nM PKI 0 min (130), 5 min (112), 10 min (82); 200 nM PKI + 0.1 μM cAMP 0 min (125), 5 min (162), 10 min (70).

Supplementary Fig S1a: control (28), 0.25 mM (30), 0.5 mM (22), 1 mM (20), 1.5 mM (18), 2 mM (17). Figure S1b: control (25), 0.25 mM (10), 0.5 mM (13), 1 mM (15), 2 mM (14). Figure S1c: control (34), 0.0 mM (25), 0.125 (11), 0.25 (22), 0.5 mM (22), 0.75 mM (29), 1 mM (24). Figure S1d: control (14), 0.5 mM (10), 1 mM (14), 2 mM (15); Fig S1e: control (25), 0.05% (14), 0.1% (13), 0.2% (12). Supplementary Fig. S2a–d: Live (53), LRP (57). Supplementary Fig. S3a: LRP 0 min (272), 5 min (237), 10 min (247); 0.1% DMSO 0 min (243), 5 min (189), 10 min (173); 6.25 μM DPD 0 min (298), 5 min (285), 10 min (271); 12.5 μM DPD 0 min (293), 5 min (247), 10 min (228); 25 μM DPD 0 min (319), 5 min (294), 10 min (273); 50 μM DPD 0 min (326), 5 min (307), 10 min (267); 100 μM DPD 0 min (311), 5 min (278), 10 min (291). Figure S3b: LRP 0 min (199), 5 min (157), 10 min (141); 0.1% DMSO 0 min (166), 5 min (117), 10 min (94); 6.25 μM DPD 0 min (218), 5 min (174), 10 min (131); 12.5 μM DPD 0 min (204), 5 min (152), 10 min (107); 25 μM DPD 0 min (218), 5 min (149), 10 min (118); 50 μM DPD 0 min (220), 5 min (173), 10 min (126); 100 μM DPD 0 min (207), 5 min (159), 10 min (144). Figure S3c: LRP 0 min (205), 5 min (211), 10 min (274); 0.1% DMSO 0 min (263), 5 min (225), 10 min (209); 0.1% DMSO + 0.1 μM cAMP 0 min (323), 5 min (233), 10 min (243); 25 μM DPD 0 min (240), 5 min (287), 10 min (274); 25 μM DPD + 0.1 μM cAMP 0 min (307), 5 min (262), 10 min (256). Figure S3d: DMSO (179), 25 μM DPD (192). Figure S3e: LRP 0 min (236), 5 min (187), 10 min (198); 0.1 μM cAMP 0 min (263), 5 min (273), 10 min (276); 135 nM H89 0 min (179), 5 min (164), 10 min (188); 135 nM H89 + 0.1 μM cAMP 0 min (193), 5 min (189), 10 min (229); 10 μM H89 0 min (193), 5 min (195), 10 min (168); 10 μM H89 + 0.1 μM cAMP 0 min (177), 5 min (175), 10 min (226). Figure S3f: LRP 0 min (424), 5 min (375), 10 min (454); 0.1 μM cAMP 0 min (408), 5 min (611), 10 min (456); 100 nM PKI 0 min (151), 5 min (137), 10 min (147); 100 nM PKI + 0.1 μM cAMP 0 min (128), 5 min (175), 10 min (216); 200 nM PKI 0 min (226), 5 min (213), 10 min (208); 200 nM PKI + 0.1 μM cAMP 0 min (213), 5 min (291), 10 min (203).

### Data Availability

All relevant data are within the manuscript and supporting information files.

## Additional Information

**How to cite this article**: Mukhopadhyay, A. G. and Dey, C. S. Reactivation of flagellar motility in demembranated *Leishmania* reveals role of cAMP in flagellar wave reversal to ciliary waveform. *Sci. Rep.* **6**, 37308; doi: 10.1038/srep37308 (2016).

**Publisher’s note**: Springer Nature remains neutral with regard to jurisdictional claims in published maps and institutional affiliations.

## Supplementary Material

Supplementary Information

Supplementary Video 1

Supplementary Video 2

Supplementary Video 3

Supplementary Video 4

Supplementary Video 5

Supplementary Video 6

Supplementary Video 7

Supplementary Video 8

## Figures and Tables

**Table 1:** Comparison of beat parameters for flagellar (tip-to-base) beating of live, LRP and basic protocol reactivated flagella of *L. donovani.*

	Percent Motility	Amplitude (μm)	Beat Frequency (Hz)	Wavelength (μm)	Flagellum Length (μm)	Wave Propagation Speed (μm/s)
Live	90.40 ± 1.78	2.35 ± 0.04	20.80 ± 0.57	10.92 ± 0.17	15.20 ± 0.34	225.98 ± 6.36
LRP reactivated	77.37 ± 2.62	2.02 ± 0.04	19.29 ± 0.65	9.95 ± 0.15	14.59 ± 0.30	192.73 ± 7.56
Basic protocol reactivated	60.24 ± 8.30	1.88 ± 0.05	13.88 ± 0.46	9.43 ± 0.18	14.24 ± 0.32	131.32 ± 5.25

Mean parameters ± SEM for flagellar beating of *L. donovani.* Five independent experiments were performed. 53, 57 and 50 cells were quantified for beat parameters of live, LRP reactivated and basic protocol reactivated cells respectively.

**Table 2:** Beat Parameters for Ciliary (base-to-tip) beating of LRP reactivated *L. donovani* in presence of 0.5 μM cAMP.

Change in Direction (degree/beat)	Amplitude (μm)	Beat Frequency (Hz)	Wavelength (μm)	Wave Propagation Speed (μm/s)	Flagellum Length (μm)
30.86 ± 3.23	1.15 ± 0.02	4.07 ± 0.21	5.37 ± 0.12	22.00 ± 1.28	14.83 ± 0.40

Mean parameters ± SEM; n = 45. Four independent experiments were performed.
