# Supplementary material for: Reactivation of flagellar motility in demembranated Leishmania reveals role of cAMP in flagellar wave reversal to ciliary waveform
Source: Sci Rep. 2016 Nov 16;6:37308. doi: 10.1038/srep37308 (PMC5110981; doi:10.1038/srep37308)
Supplement: Supplementary Information [file srep37308-s1.pdf]

<u>Supplementary Information</u>

<u>Manuscript Ref. No.; SREP-16-26913A</u>

Reactivation of flagellar motility in demembranated *Leishmania* reveals role of cAMP in flagellar wave reversal to ciliary waveform

Aakash Gautam Mukhopadhyay and Chinmoy Sankar Dey

Supplementary Figure Legends

Fig. S1. Standardization of *Leishmania* Reactivation Protocol. (**a**) Effect of different concentrations of Mg-ATP on beat frequency of reactivated *L. donovani* flagella. 1 mM Mg-ATP was used in the basic protocol. Four independent experiments were performed. (**b**) Effect of different concentrations of EGTA on beat frequency of reactivated *L. donovani* flagella. 0.5 mM EGTA was used in the basic protocol. Three independent experiments were performed. (**c**) Effect of different concentrations of EDTA on beat frequency of reactivated *L. donovani* flagella. Basic protocol did not use EDTA. Four independent experiments were performed. (**d**) Effect of different concentrations of DTT on beat frequency of reactivated *L. donovani* flagella. Three independent experiments were performed. (e) Effect of different concentrations of Triton X-100 on beat frequency of reactivated *L. donovani*. Three independent experiments were performed. Dark grey bar denotes the concentration that showed significant increase in beat frequency under our conditions. Values are mean ± SEM. Control denotes live (non-demembranated) cells. * $p < 0.05$, *** $p < 0.001$.

Fig. S2. Comparison of relationships between beat parameters of flagellar beating of live and LRP reactivated *L. donovani*. (**a**) Beat frequency vs flagellum length (**b**) Wavelength vs beat frequency (**c**) Wavelength vs flagellum length (**d**) Amplitude vs flagellum length. Solid line shows trendline for live cells. Dotted line shows trendline for

LRP reactivated cells. R^2 value denotes correlation coefficient. Black and red R^2 values are for live and LRP reactivated parasites respectively.

Fig. S3. (**a**) Effect of different concentrations of dipyridamole on percent motility of reactivated *L. donovani* over 0, 5 and 10 min. (**b**) Effect of different concentrations of dipyridamole vs proportions of flagellar and ciliary cells over 0, 5 and 10 min. Red bars denote proportion of motile cells with ciliary waveform. Blue bars denote proportion of motile cells with flagellar waveform. All experiments were performed three times. (**c**) Percent motility of cells vs time of incubation of cells. Data obtained from Fig. 5a. (**d**) Percent motility of cells incubated in DMSO or 25 µM DPD. (**e**) Percent motility of cells vs time of incubation of cells in H89. Data obtained from Fig. 6a. (**f**) Percent motility of cells vs time of incubation of cells in PKI. Data obtained from Fig. 6b.Values are mean ± SEM. * $p < 0.05$, ** $p < 0.01$, *** $p < 0.001$.

Supplementary Video Legends

Video 1. 0.1% Triton-extracted/demembranated *Leishmania donovani* cells. All of the cells are immotile, floating in the medium. Playback speed of video: 1/20 × real time (10 frames per second). Bar, 50 µm.

Video 2. Fast-capture video of immotile Triton-extracted/demembranated *Leishmania donovani*. Image acquisition speed of camera was 200 frames per second. Playback speed of video: 1/20 × real time (10 frames per second). Bar, 10 µm.

Video 3. Triton-extracted/demembranated, ATP-reactivated *Leishmania donovani* cells. The reactivated cells exhibit directional swimming, with flagella moving ahead of the cell body. Playback speed of video: 1/20 × real time (10 frames per second). Bar, 50 µm.

Video 4. Triton-extracted/demembranated *Leishmania donovani* cells followed by ATP-reactivation. Demembranated cells are immotile and floating at the beginning of the video. ATP was added (from one side of the microscopic field very gently to avoid vibration and allowed sometime to diffuse through the sample) to a final concentration of 1.5 mM at frame labelled at ~ 6s of video (white arrow). The cells were reactivated after ~ 2s, displaying directional swimming by flagellar beating. Playback speed of video: 1/20 × real time (10 frames per second). Bar, 10 µm.

Video 5. Fast-capture video of a single live *Leishmania donovani*. Flagellar waves are initiated from the tip. Few waves initiate at points other than the tip. Image acquisition speed of camera was 200 frames per second. Playback speed of video: 1/20 × real time (10 frames per second). Bar, 10 µm.

Video 6. Fast-capture video of motile Triton-extracted/demembranated, ATP-reactivated *Leishmania donovani*. Flagellar waves are initiated from the tip. Image acquisition speed of

camera was 200 frames per second. Playback speed of video: 1/20 × real time (10 frames per second). Bar, 10 µm.

Video 7. Fast-capture video of a motile Triton-extracted, ATP-reactivated *Leishmania donovani* in presence of 0.5 µM cAMP displaying ciliary (base-to-tip) waves. Video was captured immediately after addition of cAMP. The cell keeps rotating. In the video a total of ~ 14 beat cycles are completed to rotate > 360°. Emerging white arrows show points of initiation of the base-to-tip ciliary wave. Image acquisition speed of camera was 200 frames per second. Playback speed of video: 1/2 × real time (100 frames per second). Bar, 10 µm.

Video 8. Fast-capture video of motile live *Leishmania donovani* displaying ciliary (base-to-tip) waves. The cell reorients itself by generating ciliary (base-to-tip) waves (white arrows). Once the cell has altered its direction, flagellar (tip-to-base) beating takes over. A transition phase between ciliary and flagellar beating take place where the flagella extends straight like a "spear" (black arrow). Image acquisition speed of camera was 200 frames per second. Playback speed of video: 1/2 × real time (100 frames per second). Bar, 10 µm.

Supplementary Table

Table S1: Comparison of beat parameters for flagellar beating of live, LRP reactivated (without detergent and cytosol) and LRP reactivated (with detergent and cytosol) *L. donovani.*

	Percent Motility	Amplitude (μm)	Beat Frequency (Hz)	Wavelength (μm)	Wave Propagation Speed (μm/s)	Flagellum Length (μm)
**Live**	90.40 ± 1.78	2.35 ± 0.04	20.80 ± 0.57	10.92 ± 0.17	225.98 ± 6.36	15.20 ± 0.34
**LRP reactivated (detergent, cytosol free)**	70.83 ± 1.31	1.77 ± 0.06	17.77 ± 0.35	9.52 ± 0.21	168.98 ± 5.04	14.36 ± 0.39
**LRP reactivated**	77.37 ± 2.62	2.02 ± 0.04	19.29 ± 0.65	9.95 ± 0.15	192.73 ± 7.56	14.59 ± 0.30

Mean parameters ± SEM for flagellar beating of *L. donovani.* Five independent experiments were performed for live and LRP reactivated cells. Three independent experiments were performed for LRP reactivated cells without detergent and cytosol. 53, 35 and 50 cells were quantified for beat parameters of live, LRP reactivated without detergent and cytosol and LRP reactivated with detergent and cytosol cells respectively.

Supplementary Figures

Fig. S1

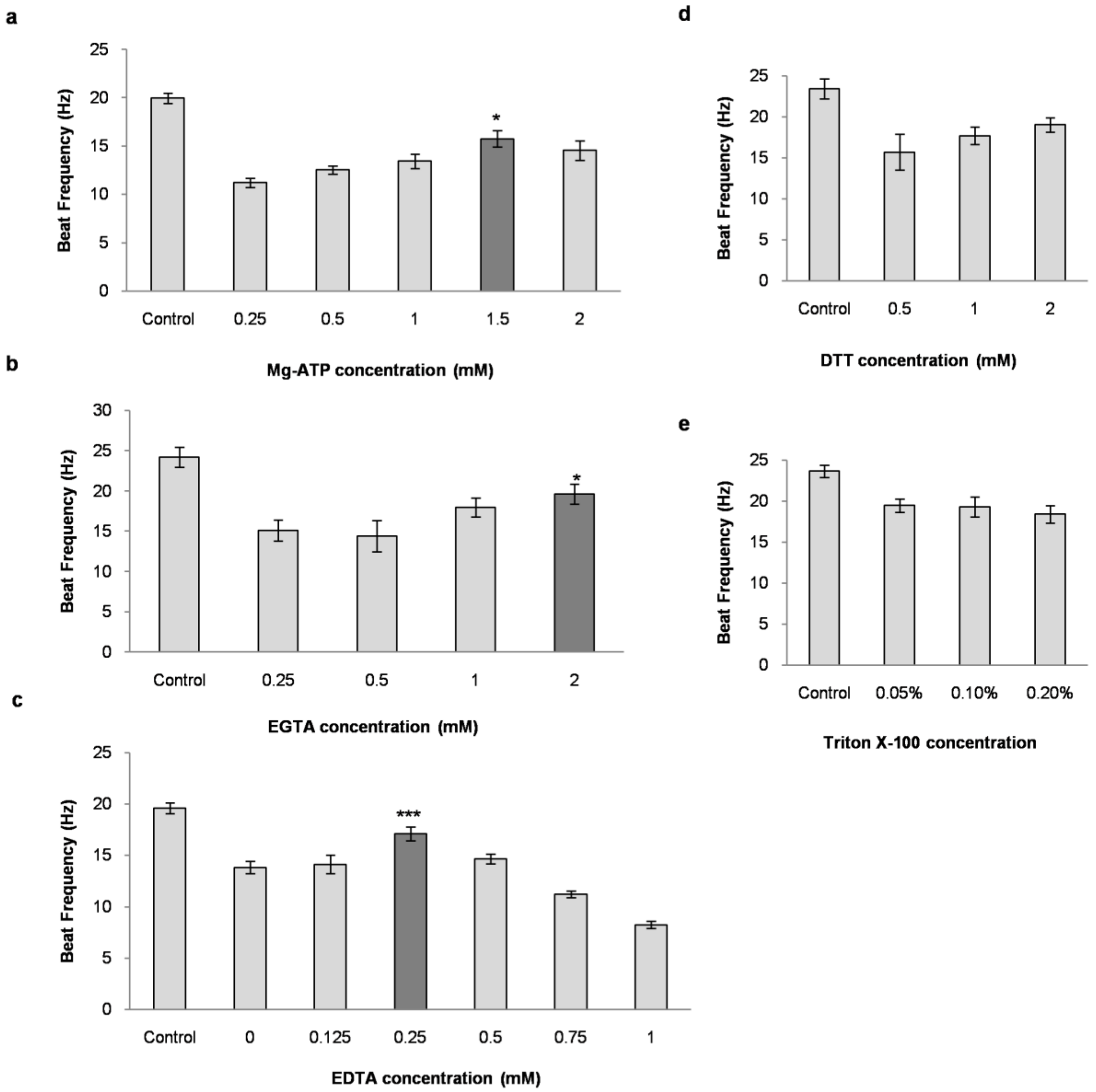

Fig. S2

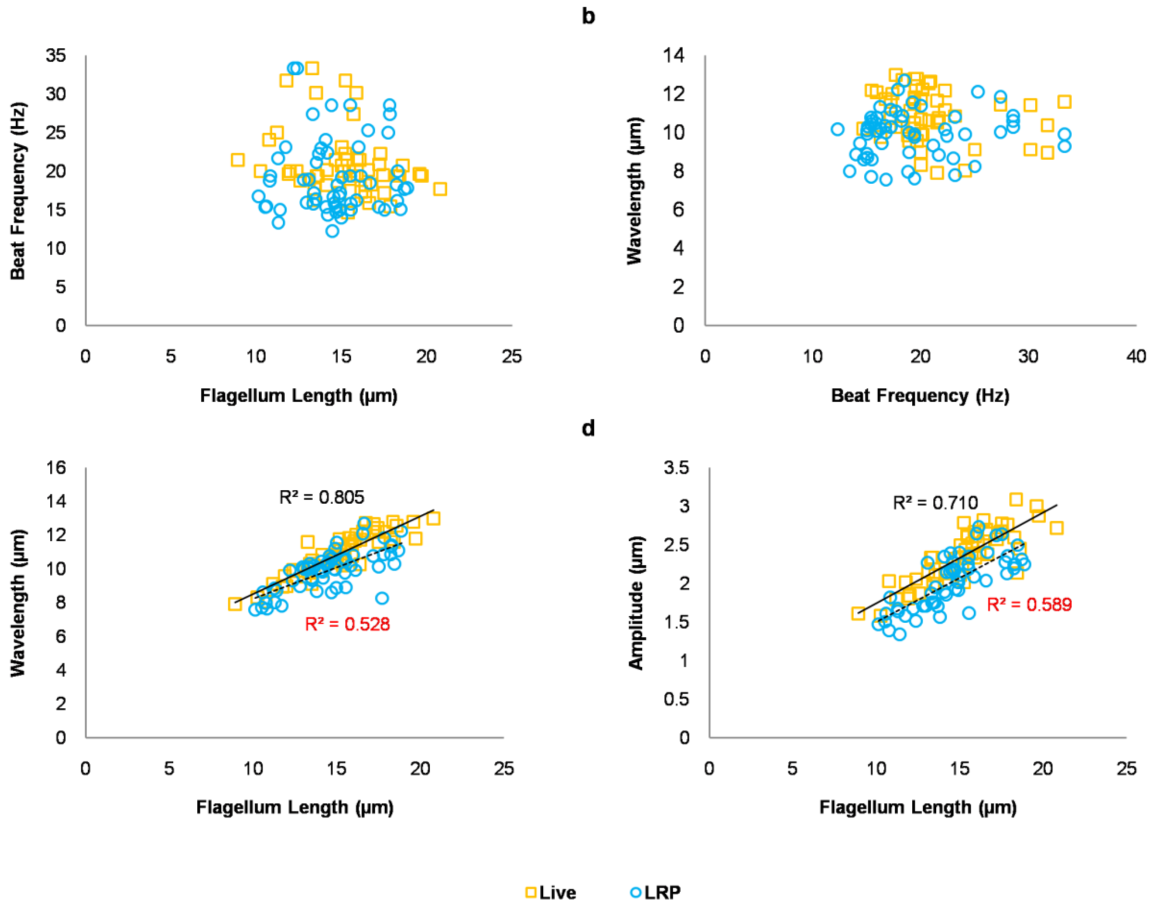
a
Beat Frequency (Hz)
Flagellum Length (µm)
b
Wavelength (µm)
Beat Frequency (Hz)
c
Wavelength (µm)
Flagellum Length (µm)
R² = 0.805
R² = 0.528
d
Amplitude (µm)
Flagellum Length (µm)
R² = 0.710
R² = 0.589
Live
LRP

Fig. S3

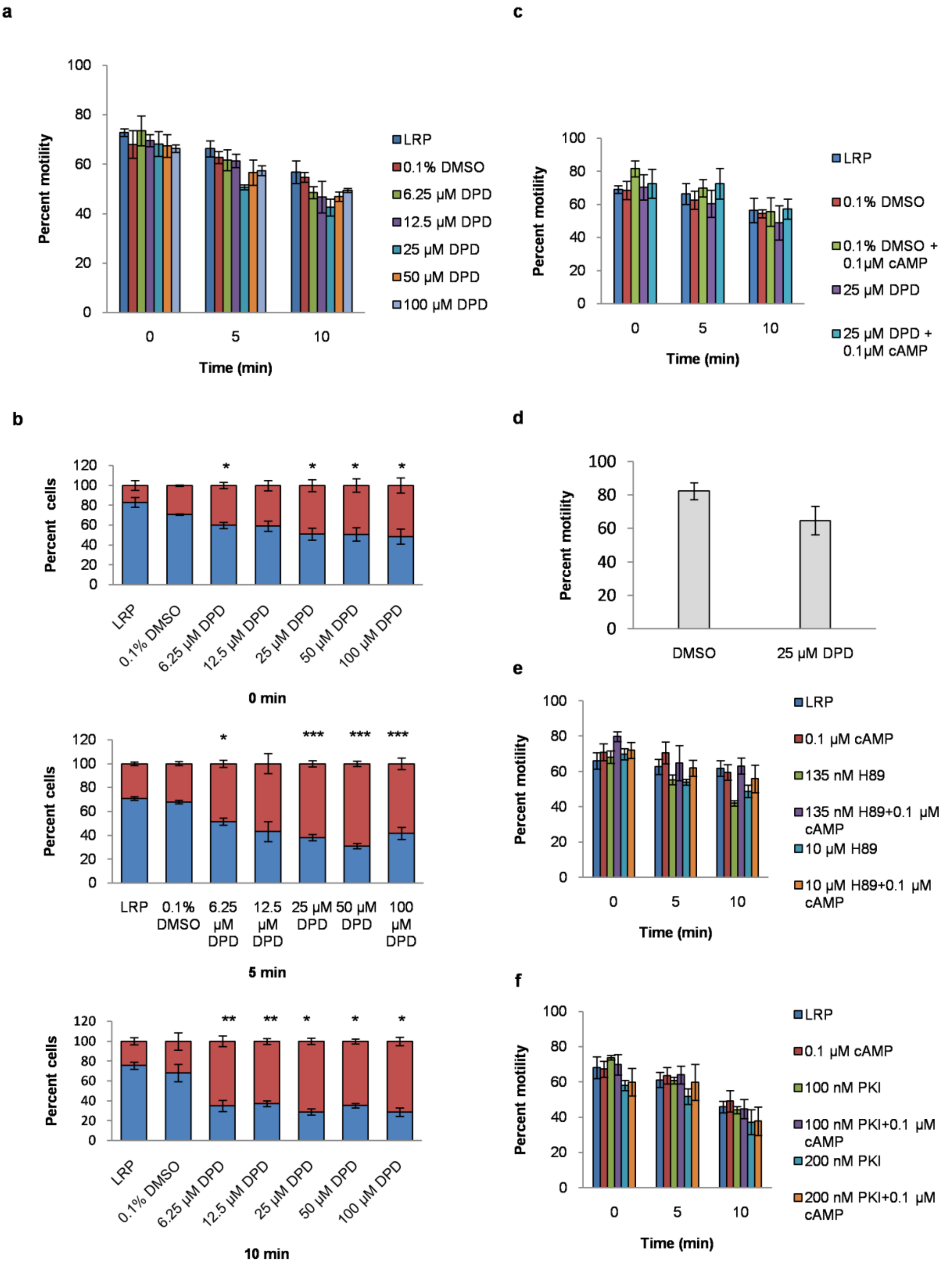
a
Percent motility
100
80
60
40
20
0
0
5
10
Time (min)
LRP
0.1% DMSO
6.25 µM DPD
12.5 µM DPD
25 µM DPD
50 µM DPD
100 µM DPD
b
Percent cells
120
100
80
60
40
20
0
*
*
*
*
LRP
0.1% DMSO
6.25 µM DPD
12.5 µM DPD
25 µM DPD
50 µM DPD
100 µM DPD
0 min
*
***
***
***
LRP
0.1% DMSO
6.25 µM DPD
12.5 µM DPD
25 µM DPD
50 µM DPD
100 µM DPD
5 min
**
**
*
*
*
10 min
c
Percent motility
Time (min)
LRP
0.1% DMSO
0.1% DMSO + 0.1µM cAMP
25 µM DPD
25 µM DPD + 0.1µM cAMP
d
Percent motility
DMSO
25 µM DPD
e
Percent motility
Time (min)
LRP
0.1 µM cAMP
135 nM H89
135 nM H89+0.1 µM cAMP
10 µM H89
10 µM H89+0.1 µM cAMP
f
Percent motility
Time (min)
LRP
0.1 µM cAMP
100 nM PKI
100 nM PKI+0.1 µM cAMP
200 nM PKI
200 nM PKI+0.1 µM cAMP
